# Higher estimated dietary intake of live microbes is associated with lower mortality in US adults

**DOI:** 10.3389/fnut.2025.1514500

**Published:** 2025-03-25

**Authors:** Xuna Liu, Yiwen Wang

**Affiliations:** ^1^Shaanxi Provincial People's Hospital, Xi’an, China; ^2^Xi'an International Medical Center Hospital Affiliated to Northwest University, Xi’an, China

**Keywords:** dietary intake of live microbes, all-cause mortality, cardiovascular mortality, NHANES, prospective study

## Abstract

**Background:**

Few studies have discussed the health benefits of total dietary intake of live microbes (TDIIM). We investigated the relationship between daily estimated TDIIM and mortality in US adults.

**Materials and methods:**

This cohort study included subjects ≥18 years from the 1999–2018 NHANES and their mortality data through December 31, 2019. The TDIIM counts were estimated based on a prior classification system, with foods categorized into low (<10^7 CFU/g), medium (10^7–10^10 CFU/g), and high (>10^10 CFU/g) levels of live microbes. Individual intakes were calculated by multiplying the microbial levels by the corresponding grams of food consumed. Weighted Cox regression models, Kaplan–Meier survival curves, and restricted cubic splines (RCS) were used to estimate the association between all-cause and cardiovascular (CVD) mortality and TDIIM.

**Results:**

Among 52,383 participants, during a median follow-up period of 118.75 months, a total of 7,711 deaths were recorded, of which 1,985 were CVD deaths. In the weighted Cox regression model, compared to participants with low TDIIM, those with high intake have lower risks of all-cause mortality (HR 0.91; 95% CI, 0.82–1.00; P for trend, 0.01), and CVD mortality (HR 0.77; 95% CI, 0.63–0.95; P for trend, 0.005). In the RCS analysis, the relationship between TDIIM and all-cause mortality exhibited a non-linear pattern with a gradual decline followed by a plateau at higher intakes, while a linear decreasing trend was observed with CVD mortality. Kaplan–Meier survival curves showed that participants with low TDIIM had a higher risk of all-cause mortality and CVD mortality.

**Conclusion:**

In this cohort study of US adults, a higher estimated TDIIM reduced the risk of all-cause and CVD mortality. These findings suggest that the ingestion of live microbes in the diet may be advantageous for human health.

## 1 Introduction

Probiotics are living microorganisms that, when administered in sufficient amounts, provide health benefits to the host (1). Dietary intake of microorganisms has the potential to make a positive contribution to human health and can influence intestinal microbiota and a wide range of diseases (2). The “old friend hypothesis” suggests that symbiotic or harmless microorganisms exposed to food are an essential and helpful source of microbial stimulation of the immune system (3). Daily intake of live microorganisms from the diet may arrive in the gut and integrate with the resident microbiota, thereby enhancing gut function, modulating the immune system, and reducing susceptibility to chronic conditions (4).

The relationship between whole ingested live microorganisms and human health has not been directly investigated. A previous study of different cross-national cohorts found that dairy consumption reduced the risk of death and major cardiovascular disease (CVD) events (5). A Japanese study showed that fermented soy intake was significantly negatively associated with CVD mortality and not with all-cause mortality (6). However, the surveys did not isolate the health outcomes caused by the contribution of live microbes in foods from the overall effects of these foods. Further, these studies under-assessed the sources of live microorganisms, which were derived not only from fermented foods but also from other foods, such as unpeeled, raw vegetables and fruits with microbial cell counts ranging from approximately 10^^6^ to 10^^8^ CFU/g (7–9). Basis on this, Marco et al. comprehensively estimated the amounts of live microbes consumed in the diet and classified all foods as having high (>10^^7^ CFU/g), medium (10^^4^–10^^7^ CFU/g) or low (<10^^4^ CFU/g) levels of live microbes (10).

Therefore, this study is the first to comprehensively investigate the relationship between estimated total dietary intake of live microbes and mortality, providing novel insights into the potential health benefits of dietary live microbes.

## 2 Materials and methods

### 2.1 Study design and setting

This study included participants from the nationally representative consecutive National Health and Nutrition Examination Survey (NHANES) 1998–2018 linked to the National Death Index through December 31, 2019. NHANES was approved by the National Center for Health Statistics (NCHS) ethics review committee, and all participants submitted written informed consent. All procedures for this study were conducted following relevant guidelines and regulations.^1^ Of the 101,316 subjects in the NHANES 1998–2018, individuals were excluded if (1) they were under 18 years of age (*n* = 42,112) (2), they had missing values for dietary live microbial intake (*n* = 6,717) (3), they had any missing values for the NHANES weight (*n* = 19) (4), they had missing follow-up data. (*n* = 85). Ultimately, a total of 52,383 subjects were included in this research (Supplementary Figure S1). This study adhered to the Strengthening the Reporting of Observational Studies in Epidemiology (STROBE) reporting guidelines (11).

### 2.2 Exposure ascertainment

The 24-h dietary data was linked to the USDA Food Survey Nutrition Database by the National Center for Health Statistics (NHANES) to evaluate nutrient and energy intakes (12–14). In Marco’ study, four experts assessed the number of live microbes in 48 food codes contained in 9388 subgroups of the NHANES database by drawing on references, authoritative reviews and the reported influence of food processing (e.g., pasteurization) on microbial viability, as well as consulting externally with USDA Agricultural Research Service microbiologist Fred Breidt. Foods with different viable microorganisms were classified as low (<10^^4^CFU/g), medium (10^^4^–10^^7^CFU/g), or high (>10^^7^ CFU/g). To quantitatively measure an individual’s approximate total dietary intake of live microorganisms (CFU), the microbial intakes of the low, medium, and high groups were assumed to be 10^^3^ CFU/g, 10^^6^ CFU/g, and 10^^9^ CFU/g, respectively, and then multiplied by the corresponding grams of food in each group and summed. In addition, as the order of magnitude of individual dietary intake of live microorganisms was too large, a logarithm with a base of 10 live microbial intakes was taken for subsequent analysis. Finally, the total population was divided into 3 groups of low, medium, and high according to individual total dietary live microorganisms <10^^7^ CFU,10^^7^–10^^10^ CFU, and > 10^^10^ CFU.

### 2.3 Mortality ascertainment

Information on mortality status (from baseline to 31 December 2019) was acquired from NCHS using a unique identification number for each participant. All-cause mortality is defined as death from any cause. Cardiovascular mortality was identified by codes I00-I09, I11, I13 and I20-I51 according to the International Classification of Diseases, Tenth Revision (ICD-10).

### 2.4 Covariates ascertainment

Demographic characteristics included age (18–39, 40–59, ≥60), sex (women, men), race (Mexican American, Non-Hispanic Black, Non-Hispanic White, Other Hispanic, other race-including multiracial), marital status (divorced/separated/widowed, married/living with a partner, never married, no record), education (<high school, high school, >high school, no record), and poverty-to-income ratio (PIR) (<1.3, 1.3–3.5, >3.5, no record), smoking status (never, former, now, no record), alcohol status (never, former, now, no record), and physical activity(no, yes, no record). BMI is calculated from measured height and weight and is classified as normal (<25kg/m^2^), overweight (25-30kg/m^2^), obese (≥30kg/m^2^), and no record. The estimated glomerular filtration rate (eGFR, mL/min/1.73m^2^) was categorized into <60 and ≥ 60 groups, which was calculated from serum creatinine measurements using the 2009 Chronic Kidney Disease Epidemiology Collaboration formula (15). Total diet quality was estimated using the 2015 version of the Healthy Eating Index (HEI) and total energy intake (16). Comorbidities included hypertension, diabetes, CVD, and cancer, of which the first two are diagnosed through index measurements, medication use, and self-reporting, while CVD and cancer are identified through self-reporting.

### 2.5 Statistical analysis

Individual sample weights were created using WTDR4YR weights in 1999–2002 and WTDRD1 weights in 2003–2018, given the complex sampling design of NHANES. During the baseline characteristics analysis, continuous variables were presented using weighted means (standard errors), and categorical variables were presented using sample numbers (weighted percentages). One-way ANOVA and chi-square tests were performed to compare differences according to baseline dietary intake of live microorganisms (<10^^7^ CFU,10^^7^–10^^10^ CFU, and > 10^^10^ CFU).

Weighted multivariable Cox regression analysis was used to estimate corrected risk ratios (HR) for mortality and their 95%CI based on dietary live microbial intake in the low, medium, and high groups at baseline. The crude model did not adjust for any potential confounders. Model 1 adjusted for age, sex, race, education, marital status, and PIR. Model 2 was further adjusted for HEI, total energy intake, smoking status, alcohol status, physical activity, and BMI. Model 3 additionally adjusted for hypertension, diabetes, CVD, cancer, and eGFR. We carried out survival analyses with the use of standardized Kaplan–Meier curves and Log ranking tests. The non-linear relationship between mortality and log(dietary live microbial intake) was explored by multivariable-adjusted Cox restricted cubic spline (RCS) regression models. RCS regression models were used to explore the potential non-linear relationship between TDIIM and mortality outcomes, allowing for a more flexible assessment of the association by fitting a smooth curve to the data. Subgroup analyses were undertaken by sex (female, male), age (18–39, 40–59, ≥60), BMI (normal, overweight, obese, no record), smoking status (never, former, now, no record), alcohol status (never, former, now, no record), physical activity(no, yes, no record), hypertension (no, yes), diabetes (no, boundary, yes), CVD (no, yes), and eGFR (<60,≥60, no record). Multiplicative interactions were used to estimate potential interactions between multiple subgroup elements and log(dietary live microbial intake).

Sensitivity analyses were conducted to verify the robustness of the study results. Firstly, participants who died within 2 years of follow-up were excluded to eliminate potential reverse causality. Secondly, participants with chronic diseases (including hypertension, cardiovascular disease, diabetes mellitus, cancer, and eGFR<60) were excluded, as these individuals were more likely to die during the follow-up period. Thirdly, to test the sensitivity of dietary live microbial intake to the findings, we re-quantified the total intake of live microbes (CFU) in individuals’ diets. Microbial intakes were assumed to be 10^^3.5^ CFU/g, 10^^7^ CFU/g, and 10^^10^ CFU/g for the low, medium, and high groups, respectively, then multiplied by the number of grams of food in each group and summed, and later re-classified into low, medium, and high groups by <10^^8^ CFU, 10^^8^–10^^11^ CFU, and > 10^^11^ CFU for analysis. In addition, the newly calculated logarithms of dietary live microbial intake were analyzed with different mortality rates. Finally, the association with the dietary intake of live microorganisms was assessed using mortality due to accidents and injuries as the dependent variable.

All statistical analyses were performed using R version 4.2.1 (R Foundation for Statistical Computing, Vienna, Austria; http://www.r-project.org), and statistical significance was ascertained by a two-sided *p* value <0.05.

## 3 Results

### 3.1 Baseline characteristics

A total of 52,383 subjects, 51.62% of whom were female, with a mean age of 46.10 ± 0.20 years, were ultimately enrolled in our study. Over a median follow-up period of 118.75 months, there were 7,711 deaths and an all-cause mortality rate of 14.72%, including 1985 CVD deaths and a CVD mortality rate of 3.79%. The mean individual estimated dietary live microbial intake was 20,623.09 ± 536.25 (*10^^6^ CFU) and the mean of its logarithm was 8.14 ± 0.02. Of these, the low, medium, and high dietary live microbial intake groups had 19,917, 22,981, and 9,485 participants, respectively. According to Table 1, participants with higher levels of dietary intake of live microorganisms were more likely to be female, Non-Hispanic White, married/living with a partner, never smokers, current drinkers, physical exercises, had higher levels of education, wealth, BMI, eGFR, HEI, and energy intake, and had no comorbid hypertension, diabetes, cardiovascular disease, or cancer (all *p* < 0.0001). Finally, the higher the dietary intake of live microorganisms, the fewer deaths there were in the group (*p* < 0.0001).

**Table 1:** Demographic characteristics according to the dietary intake of live microbes.

	Total	Low	Medium	High	P value
		<10^^7^ CFU	10^^7^–10^^10^ CFU	>10^^10^ CFU	
No. of participants	52,383	19,917(38.02)	22,981(43.87)	9,485(18.11)	
Age, y, mean (SE)	46.10(0.20)	44.29(0.21)	47.65(0.24)	45.93(0.31)	< 0.0001
Age, *n* (%)					< 0.0001
18–39	20,456(39.05)	8,301(43.84)	8,263(36.65)	3,892(39.50)	
40–59	15,421(29.44)	5,670(34.61)	6,888(35.91)	2,863(36.74)	
≥60	16,506(31.51)	5,946(21.55)	7,830(27.44)	2,730(23.77)	
Sex, *n* (%)					< 0.0001
Female	27,040(51.62)	9,639(48.29)	12,154(53.22)	5,247(54.21)	
Male	25,343(48.38)	10,278(51.71)	10,827(46.78)	4,238(45.79)	
Race, *n* (%)					< 0.0001
Mexican American	9,644(18.41)	3,193(7.95)	5,049(10.07)	1,402(5.97)	
Non-Hispanic Black	11,226(21.43)	5,820(16.83)	4,142(9.72)	1,264(6.10)	
Non-Hispanic White	22,854(43.63)	7,631(62.02)	10,049(67.89)	5,174(77.35)	
Other Hispanic	4,202(8.02)	1,590(5.99)	1830(5.37)	782(4.58)	
Other Race	4,457(8.51)	1,683(7.21)	1911(6.95)	863(6.00)	
Marital status, *n* (%)					< 0.0001
Divorced/separated/widowed	10,735(20.49)	4,393(19.77)	4,649(17.93)	1,693(15.71)	
Married/living with a partner	29,228(55.8)	10,060(53.92)	13,506(62.03)	5,662(63.87)	
Never married	10,284(19.63)	4,451(22.14)	4,019(17.11)	1814(17.75)	
No record	2,136(4.08)	1,013(4.17)	807(2.93)	316(2.67)	
Education level, *n* (%)					< 0.0001
<High school	6,013(11.48)	2,519(7.15)	2,829(5.92)	665(2.98)	
High school	21,174(40.42)	9,257(44.92)	8,858(34.52)	3,059(28.20)	
>High school	25,134(47.98)	8,110(47.84)	11,270(59.49)	5,754(68.77)	
No record	62(0.12)	31(0.08)	24(0.07)	7(0.05)	
Poverty-to-income ratio, *n* (%)					< 0.0001
<1.3	15,290(29.19)	6,920(27.49)	6,208(18.89)	2,162(15.06)	
1.3–3.5	18,213(34.77)	7,048(35.35)	8,068(32.92)	3,097(29.87)	
>3.5	14,420(27.53)	4,233(30.00)	6,692(41.00)	3,495(48.55)	
No record	4,460(8.51)	1716(7.16)	2013(7.20)	731(6.52)	
BMI, *n* (%)					< 0.0001
Under/normal weight	16,165(30.86)	6,089(30.48)	7,007(31.89)	3,069(33.17)	
Overweight	17,048(32.54)	6,147(30.52)	7,736(33.46)	3,165(33.62)	
Obese	18,345(35.02)	7,306(37.35)	7,903(33.59)	3,136(32.27)	
No record	825(1.57)	375(1.65)	335(1.07)	115(0.94)	
Smoking status, *n* (%)					< 0.0001
Never	27,046(51.63)	9,520(47.76)	12,265(54.15)	5,261(56.37)	
Former	12,158(23.21)	4,151(21.28)	5,727(25.82)	2,280(24.93)	
Now	10,210(19.49)	4,902(27.56)	3,812(17.73)	1,496(16.72)	
No record	2,969(5.67)	1,344(3.40)	1,177(2.29)	448(1.98)	
Alcohol status, *n* (%)					< 0.0001
Never	6,969(13.3)	2,749(11.65)	3,138(11.34)	1,082(9.07)	
Former	8,086(15.44)	3,393(14.95)	3,522(13.06)	1,171(10.65)	
Now	30,923(59.03)	11,100(63.61)	13,627(67.26)	6,196(73.03)	
No record	6,405(12.23)	2,675(9.79)	2,694(8.34)	1,036(7.25)	
Physical activity, *n* (%)					< 0.0001
No	16,820(32.11)	7,289(33.70)	6,796(26.13)	2,735(25.14)	
Yes	15,531(29.65)	5,207(28.33)	6,752(33.64)	3,572(43.46)	
No record	20,032(38.24)	7,421(37.97)	9,433(40.23)	3,178(31.40)	
eGFR, mL/min/1.73m2, *n* (%)					< 0.0001
<60	4,202(8.02)	1,651(6.42)	1925(6.93)	626(5.25)	
≥60	45,250(86.38)	16,950(87.66)	19,906(88.65)	8,394(90.86)	
No record	2,931(5.6)	1,316(5.93)	1,150(4.42)	465(3.89)	
DILM,(*10^^6^CFU),mean (SE)	20623.09(536.25)	3.16(0.02)	573.03(16.49)	91313.73(1453.90)	< 0.0001
Log-DILM, mean (SE)	8.14(0.02)	6.44(0.00)	8.16(0.01)	10.76(0.01)	< 0.0001
HEI, mean (SE)	50.42(0.18)	45.35(0.17)	53.10(0.19)	53.13(0.25)	< 0.0001
Total energy intake, kcal, mean (SE)	2166.62(6.85)	2063.51(9.18)	2169.97(9.81)	2320.55(14.15)	< 0.0001
Hypertension, n (%)					< 0.0001
No	31,638(60.4)	11,849(63.10)	13,720(62.75)	6,069(66.38)	
Yes	20,745(39.6)	8,068(36.90)	9,261(37.25)	3,416(33.62)	
DM, *n* (%)					< 0.0001
Boundary	3,400(6.49)	1,305(6.22)	1,500(6.23)	595(5.97)	
No	40,594(77.49)	15,368(81.29)	17,615(80.59)	7,611(83.40)	
Yes	8,389(16.01)	3,244(12.50)	3,866(13.19)	1,279(10.63)	
CVD, *n* (%)					< 0.0001
No	46,886(89.51)	17,640(90.28)	20,562(91.31)	8,684(93.00)	
Yes	5,497(10.49)	2,277(9.72)	2,419(8.69)	801(7.00)	
Cancer, *n* (%)					< 0.0001
No	47,833(91.31)	18,433(92.31)	20,837(89.92)	8,563(89.51)	
Yes	4,550(8.69)	1,484(7.69)	2,144(10.08)	922(10.49)	
Status, *n* (%)					< 0.0001
Alive	44,672(85.28)	16,785(87.46)	19,444(88.10)	8,443(91.86)	
Death	7,711(14.72)	3,132(12.54)	3,537(11.90)	1,042(8.14)	

eGFR, Estimated glomerular filtration rate; DILM, Dietary Intake of Live Microbes; HEI, healthy eating index; DM, diabetes mellitus; CVD, cardiovascular disease.

### 3.2 Estimated dietary live microbial intake and all-cause and cardiovascular mortality

The results of the COX regression analysis were displayed in Table 2. A dose–response relationship was found between dietary live microbial intake and all-cause mortality and CVD mortality. In the all-cause mortality analysis, compared to the low dietary live microbial intake group, the multivariate-adjusted HRs were smaller in the high group for model 1 (HR = 0.81, 95% CI = 0.73–0.90, P for trend<0.0001), model 2 (HR = 0.89, 95% CI = 0.80–0.99, P for trend = 0.01) and model 3 (HR = 0.91. 95% CI = 0.82–1.00, P for trend = 0.01). In addition, after multivariate adjustment, log (dietary intake of live microbes) was significantly associated with a reduced risk of all-cause mortality (HR = 0.97, 95% CI = 0.95–0.99). For the CVD mortality analysis, after adjustment for all confounding variables, the HR for microbial intake was smaller in the high group (HR = 0.77, 95% CI 0.63–0.95, P for trend = 0.005) and the log (dietary intake of live microbes) was negatively associated with CVD mortality (HR = 0.93, 95% CI = 0.89–0.97).

**Table 2:** Weighted multivariable cox regression on dietary intake of live microbes and mortality.

	All-cause mortality				CVD mortality			
	Crude model	Model 1	Model 2	Model 3	Crude model	Model 1	Model 2	Model 3
Low	Ref	Ref	Ref	Ref	Ref	Ref	Ref	Ref
Medium	0.91(0.85,0.97)	0.84(0.79,0.90)	0.90(0.85,0.96)	0.91(0.85,0.97)	0.87(0.77,0.98)	0.81(0.71,0.92)	0.85(0.76,0.96)	0.85(0.73,0.98)
High	0.70(0.63,0.77)	0.81(0.73,0.90)	0.89(0.80,0.99)	0.91(0.82,1.00)	0.58(0.47,0.71)	0.69(0.56,0.84)	0.78(0.64,0.95)	0.77(0.63,0.95)
P for trend	<0.0001	<0.0001	0.01	0.01	<0.0001	<0.0001	0.004	0.005
Log-DILM	0.91(0.89,0.93)	0.94(0.92,0.96)	0.96(0.94,0.99)	0.97(0.95,0.99)	0.87(0.83,0.90)	0.89(0.86,0.93)	0.93(0.89,0.97)	0.93(0.89,0.97)

Log-DILM: Log-(dietary intake of live microbes). Data are presented as HR (95%CI). Crude Model: unadjusted model. Model 1: adjust for age, sex, race, education, marital status, and poverty-to-income ratio. Model 2: adjust additionally for Healthy Eating Index, total energy intake, smoking status, alcohol status, physical activity, and BMI. Model 3: adjust additionally for hypertension, diabetes, CVD, cancer, and eGFR. Dietary microbial counts were divided into three groups: low, medium, and high, with values of < 10^^7^ CFU, 10^^7^–10^^10^ CFU, and > 10^^10^ CFU, respectively.

**Figure 1:**
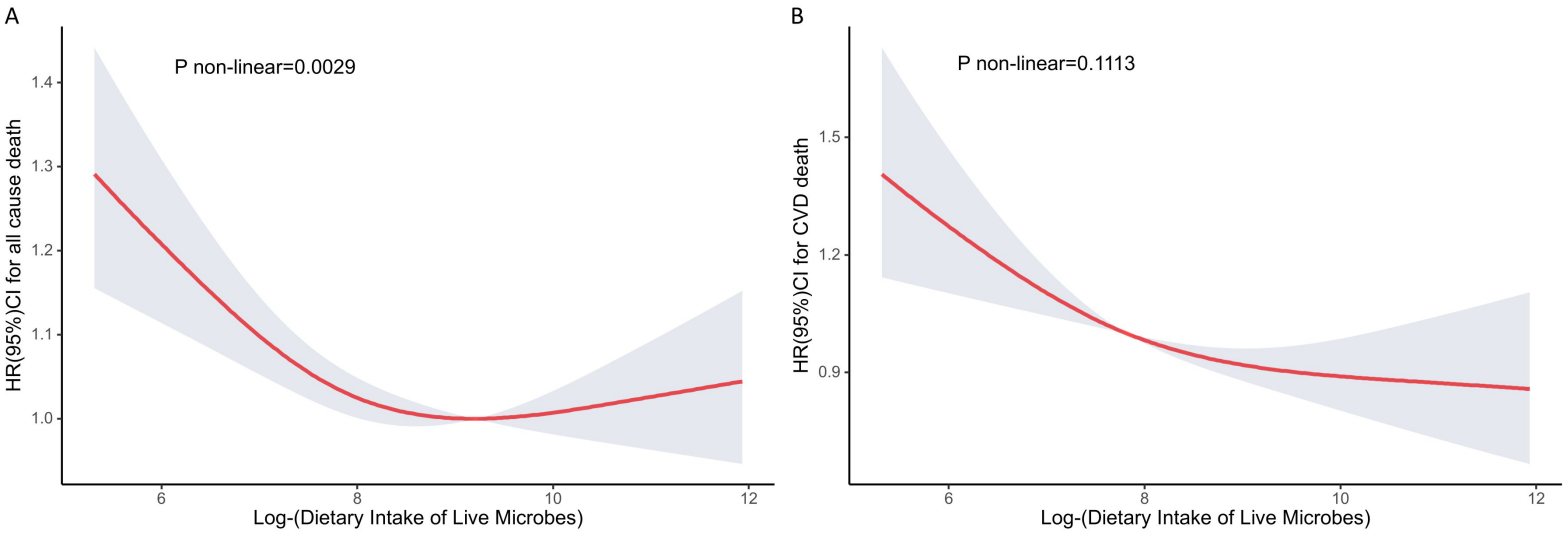
Analysis of restricted cubic spline regression for all-cause **(A)** and cardiovascular disease **(B)** death by dietary intake of live microbes.* Adjusted restricted cubic spline models adjusted for age, sex, race, education, marital status, poverty-to-income ratio, Healthy Eating Index, total energy intake, smoking status, alcohol status, physical activity, BMI, hypertension, diabetes, CVD, cancer, eGFR.

In restricted cubic spline regression models fully adjusted for covariates, the relationship between log dietary live microbial intake and mortality was non-linear (Figure 1A, p for nonlinear = 0.0029) and the relationship with CVD mortality (Figure 1B) was linear (p for nonlinear = 0.1113), with an overall decreasing trend.

**Figure 2:**
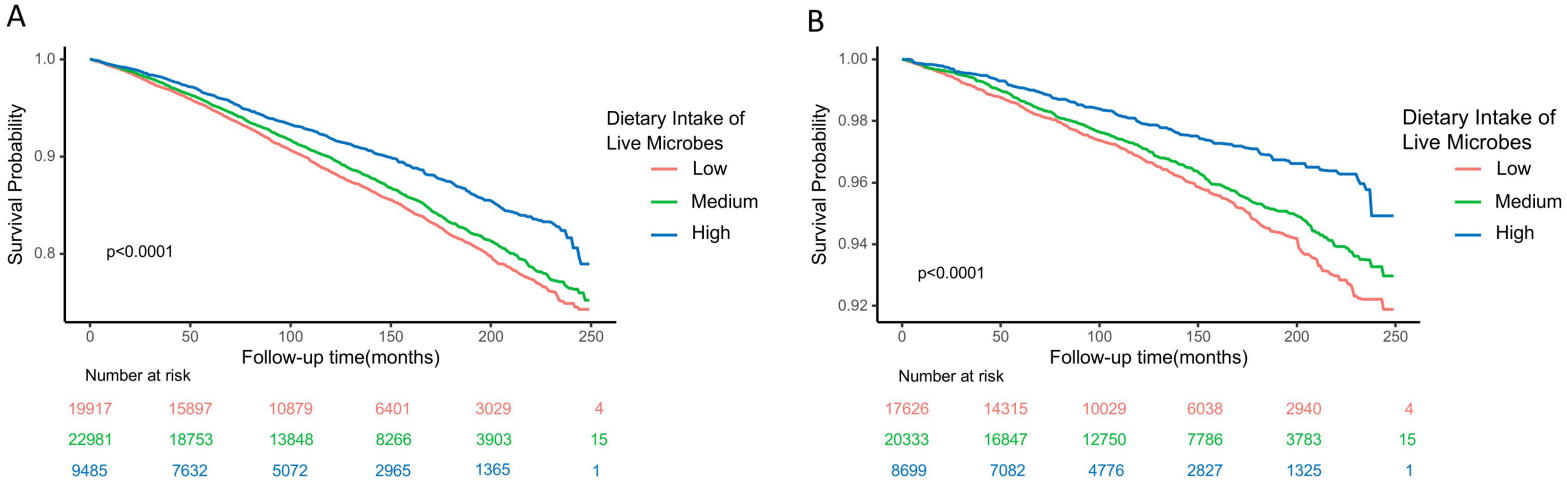
Kaplan-Meier survival curve for all-cause **(A)** and cardiovascular disease **(B)** mortality by dietary intake of live microbes. *Dietary microbial counts were divided into three groups: low, medium, and high, with values of <107 CFU, 107-1010 CFU, and >1010 CFU respectively.

As revealed by the Kaplan–Meier survival curves (Figure 2), higher dietary live microbial intake was accompanied by significantly lower all-cause mortality (Figure 2A) and CVD mortality (Figure 2B) in the following life (log-ranked *p* < 0.0001).

### 3.3 Subgroup and interaction analyses

**Figure 3:**
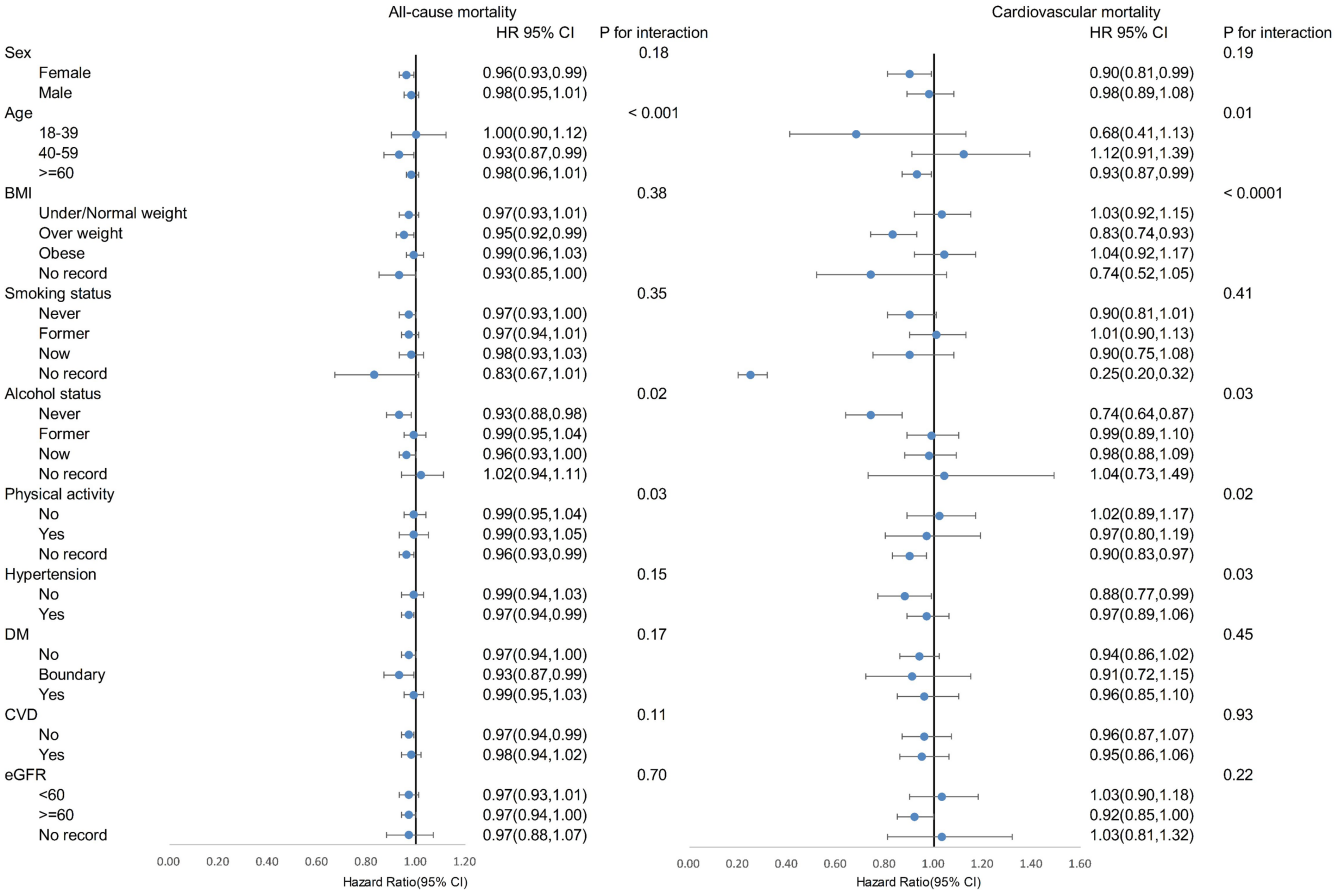
Subgroup analysis on log (dietary intake of live microbes) and mortality. *The analysis adjusted for age, sex, race, education, marital status, poverty-to-income ratio, Healthy Eating Index, total energy intake, smoking status, alcohol status, physical activity, BMI, hypertension, diabetes, CVD, cancer, and eGFR.

As illustrated in Figure 3, subgroup analyses showed the link between log(dietary live microbial intake) and reduced overall mortality was significant in females, those aged 40–59, overweight individuals, never drinkers, people with hypertension, those with borderline diabetes, and those without CVD (all *p* < 0.05). This link was also tied to lower CVD mortality in females, individuals aged ≥60, overweight people, never drinkers, and those without hypertension (all *p* < 0.05). Factors like age, alcohol status, and physical activity modified the association with all-cause mortality decline (P for interaction <0.05). Age, BMI, drinking status, physical activity, and hypertension altered the link with reduced CVD mortality (P for interaction <0.05). No significant interactions were found for other subgroups. These findings suggest that higher dietary intake of live microbes may have significant public health implications for reducing mortality risk, particularly in special populations.

### 3.4 Sensitivity analyses

In sensitivity analyses, dietary live microbial intake remained negatively associated with all-cause mortality and CVD mortality after excluding subjects dying within the first 2 years of follow-up (Supplementary Table S1). After excluding the baseline chronic disease population, dietary live microbial intake was negatively related to all-cause mortality and CVD mortality, but the relationship with all-cause mortality was not statistically significant (Supplementary Table S2), so we performed an RCS analysis to reveal that the negative association continued to exist (Supplementary Figure S2). Furthermore, these results remained statistically significant (all *p* < 0.05) after recalculation of dietary live microbial intake (Supplementary Table S3). No statistically significant associations were obtained in sensitivity analyses examining the association between dietary live microbial intake and mortality from accidents and injuries (Supplementary Table S4S1).

## 4 Discussion

To our knowledge, this is the first study to examine estimated total dietary intake of live microbes and mortality. In this nationally representative cohort study of US adults, we found that higher dietary intake of live microorganisms was associated with lower risk of all-cause and CVD mortality. Our findings were consistent across subgroup analyses and different sensitivity analyses. Notably, higher dietary live microbial intake was more protective against the risk of all-cause and CVD mortality among women, middle-aged and older adults, and those who were overweight. While our study reveals an inverse association between TDIIM and mortality, it is important to note that further intervention studies, such as randomized controlled trials, are necessary to establish causality.

The type, level of processing and origin of the food determines the type and number of microorganisms in the food, including bacteria, molds and yeasts. Fresh vegetables and fruits are found to contain a wide range of microorganisms, but usually <10^^6^ CFU/g (8), whereas fermented foods have higher levels, around 10^^8^–10^^11^ CFU/g, such as yoghurt (17). There is a wide variety of probiotics that can provide health benefits to the host, including bacterial genera such as Bifidobacterium, Lactobacillus, Lactococcus, Bacillus, Pediococcus, Enterococcus, *Escherichia coli*, Streptococcus, Propionibacterium, and Leuconostoc, and yeast or Saccharomyces (18).

Marco’ study showed that fruits, vegetables and fermented dairy products are the top 3 food groups that provide Americans with dietary live microorganisms (10). In a large international prospective cohort study, greater fruit, vegetable and legume intake was found to be associated with a reduced risk of major cardiovascular disease, myocardial infarction, cardiovascular mortality, non-cardiovascular mortality and total mortality in an analysis adjusted for age and sex (19). A study from China also showed that higher fruit, vegetable and legume consumption was associated with lower risk of cardiovascular disease mortality, cancer incidence, cancer mortality and all-cause mortality, and these associations remained significant for all-cause mortality after adjusting for additional socioeconomic and lifestyle factors (20). A meta-analysis from cohort studies has identified a reduced risk of cardiovascular disease associated with the intake of fermented dairy products (21). Dairy consumption was previously found to be associated with a lower risk of death and major cardiovascular disease events in a different cross-national cohort study (5). As a partial source of intake of live dietary microorganisms, these studies may provide indirect evidence to our study that partial dietary microbial intake is beneficial and may reduce mortality.

Subgroup analysis showed that the negative association between dietary live microbial intake and mortality was modified by age, drinking status, and basal hypertension. Overall, the subgroup analyses indicated that the protective effect of TDIIM on mortality was more evident in specific subpopulations, highlighting the potential targeted benefits of dietary live microbes. Currently, no studies have investigated the relationship between dietary live microbial intake and mortality risk and further studies are needed to validate these results.

The reduction of all-cause mortality and CVD mortality by high dietary live microorganisms may be due to the following reasons. Firstly, probiotics can cause an increase in short-chain fatty acids (e.g., butyrate), protect the integrity of the gut, regulate metabolism and reduce the inflammatory state of the organism (22, 23). Secondly, some probiotics, including *Lactobacillus acidophilus* and *Bifidobacterium bifidum*, can reduce elevated cholesterol levels, thereby facilitating the prevention and treatment of cardiovascular disease (24, 25). Thirdly, probiotics such as *L. reuteri* and *L. fermentum* can reduce pro-inflammatory cytokines and attenuate oxidative stress, thereby preventing the development and progression of atherosclerosis (26–28).

This study has several strengths. Firstly, it is the first study to assess the relationship between total dietary intake of live microorganisms and mortality. Secondly, the NHANES data were selected using a complex multi-stage probability sampling design to select a representative sample and ensure high-quality data. Third, this study controlled for many confounding factors. Fourth, this study conducted a stratified analysis and found that live microbial intake was more meaningful in reducing mortality in women, middle-aged and elderly, and overweight populations, making the findings more targeted. Therefore, the results of this study have important public health implications for the management of dietary live microbial intake.

However, the study also has some limitations. Firstly, the observational study design does not allow for a true causal relationship to be established. Second, this study is particular to the United States; elsewhere, the intake of live microorganisms may vary considerably due to differences in food types and dietary habits (e.g., fermented foods). Third, the intake of live dietary microbes in this study was based on the 3-level classification system of Marco et al. and there was only a single baseline measurement, so the precision of the measurement of live dietary microbes in this study was not very high. However, the dietary habits of most adults are fixed and difficult to change, and it is impractical to precisely define dietary viable microbial intake. At the same time, this study found the negative association between dietary live microbial intake and mortality to be sufficiently stable using different estimates, which is largely representative of the actual situation.

## 5 Conclusion

In conclusion, we found that higher dietary live microbial intake reduced the risk of all-cause and CVD mortality, and was particularly protective for women, middle-aged and older, and overweight individuals. Our study directly suggests that dietary intake of live microorganisms may have health benefits for humans. Future research could benefit from incorporating biomarkers of microbial exposure or leveraging advancements in food microbiome quantification to improve the accuracy and precision of dietary live microbe intake assessments. Conducting randomized controlled trials to confirm the causal link between TDIIM and health outcomes is vital. Validating the beneficial effects could facilitate the development of dietary guidelines emphasizing live microbial intake for disease prevention and health promotion.

## Data Availability

Publicly available datasets were analyzed in this study. This data can be found here: https://www.cdc.gov/nchs/data_access.htm.

## Footnotes

^1^ https://www.cdc.gov/nchs/data_access/restrictions.htm
