# Supplementary material for: Higher estimated dietary intake of live microbes is associated with lower mortality in US adults
Source: Front Nutr. 2025 Mar 25;12:1514500. doi: 10.3389/fnut.2025.1514500 (PMC11975568; doi:10.3389/fnut.2025.1514500)
Supplement: Supplementary file 1 [file Data_Sheet_1.docx]

Supplementary Material

Higher Estimated Dietary Intake of Live Microbes is Associated with Lower Mortality in US Adults

Xuna Liu ^1^, Yiwen Wang ^2^

^1^ Shaanxi Provincial People's Hospital, Xi’an, 710068, China.

^2^ Xi'an International Medical Center Hospital Affiliated To Northwest University, Xi’an 710119, China

*** Correspondence:**Corresponding Author: Yiwen Wang; wangyiwenmoon@stu.xjtu.edu.cn

# Supplementary Data

# Supplementary Figures and Tables

## Supplementary Figures

Participants extracted from NHANES

1999-2018 (n=101,316)

Excluded:

Participants with age <18 years (n=42,112).

Included participants (n=59,204)

Included participants (n=52,468)

Excluded:

Participants with missing values for dietary live microbial intake (n =6,717);

Participants with missing data for the NHANES weight (n = 19).

Eligible participants for analysis(n=52,383)

Excluded:

Participants with any missing follow-up data (n = 85).

**Supplementary Figure S1. Flowchart of the sample selection from NHANES 1999-2018 Fig S2. Analysis of Restricted Cubic Spline Regression excluding participants who had chronic diseases at baseline.**

**Supplementary Figure S2. Analysis of Restricted Cubic Spline Regression excluding participants who had chronic diseases at baseline.**

Adjusted restricted cubic spline models adjusted for age, sex, race, education, marital status, poverty-to-income ratio, Healthy Eating Index, total energy intake, smoking status, alcohol status, physical activity, and BMI.

## Supplementary Tables

**Supplementary Table S1. Sensitivity analysis on the relationship between dietary intake of live microbes and mortality excluding participants who died within two years of follow-up.**

|  | All-cause mortality | |  | CVD mortality | |
| --- | --- | --- | --- | --- | --- |
|  | HR (95%CI) | P value |  | HR (95%CI) | P value |
| Low | ref |  |  | ref |  |
| Medium | 0.89(0.83,0.95) | <0.001 |  | 0.83(0.71,0.98) | 0.03 |
| High | 0.89(0.80,0.99) | 0.03 |  | 0.75(0.60,0.93) | 0.01 |
| P for trend | | 0.01 |  |  | 0.004 |
|  |  |  |  |  |  |
| Log-DILM | 0.97(0.94,0.99) | 0.005 |  | 0.92(0.88,0.97) | 0.001 |

Log-DILM: Log-(dietary intake of live microbes).

Data are presented as HR (95%CI).

Model adjusted for age, sex, race, education, marital status, poverty-to-income ratio, Healthy Eating Index, total energy intake, smoking status, alcohol status, physical activity, BMI, hypertension, diabetes, CVD, cancer, and eGFR.

Dietary microbial counts were divided into three groups: low, medium, and high, with values of <10^7 CFU, 10^7-10^10 CFU, and >10^10 CFU respectively.

**Supplementary Table S2. Sensitivity analysis on the relationship between dietary intake of live microbes and mortality excluding participants who had chronic diseases at baseline.**

|  | All-cause mortality | |  | CVD mortality | |
| --- | --- | --- | --- | --- | --- |
|  | HR (95%CI) | P value |  | HR (95%CI) | P value |
| Low | ref |  |  | ref |  |
| Medium | 0.85(0.71,1.03) | 0.09 |  | 0.92(0.53, 1.60) | 0.78 |
| High | 0.85(0.65,1.11) | 0.24 |  | 0.54(0.32, 0.93) | 0.03 |
| P for trend |  | 0.16 |  |  | 0.06 |
|  |  |  |  |  |  |
| Log-DILM | 0.94(0.89,1.01) | 0.08 |  | 0.86(0.77, 0.97) | 0.01 |

Log-DILM: Log-(dietary intake of live microbes).

Data are presented as HR (95%CI).

Model adjusted for age, sex, race, education, marital status, poverty-to-income ratio, Healthy Eating Index, total energy intake, smoking status, alcohol status, physical activity, and BMI.

Dietary microbial counts were divided into three groups: low, medium, and high, with values of <10^7 CFU, 10^7-10^10 CFU, and >10^10 CFU respectively.

**Supplementary Table S3.** **Sensitivity analysis on the relationship between dietary intake of live microbes and mortality re-estimation of microbial intake.**

|  | All-cause mortality | |  | CVD mortality | |
| --- | --- | --- | --- | --- | --- |
|  | HR (95%CI) | P value |  | HR (95%CI) | P value |
| Low | ref |  |  | ref |  |
| Medium | 0.89(0.83,0.95) | 0.001 |  | 0.84(0.71,0.99) | 0.03 |
| High | 0.89(0.80,0.99) | 0.03 |  | 0.75(0.60,0.94) | 0.01 |
| P for trend | | 0.01 |  |  | 0.01 |
|  |  |  |  |  |  |
| Log-DILM | 0.97(0.95,0.99) | 0.003 |  | 0.93(0.89,0.97) | <0.001 |

Log-DILM: Log-(dietary intake of live microbes).

Data are presented as HR (95%CI).

Model adjusted for age, sex, race, education, marital status, poverty-to-income ratio, Healthy Eating Index, total energy intake, smoking status, alcohol status, physical activity, BMI, hypertension, diabetes, CVD, cancer, and eGFR.

Microbial intakes were assumed to be 10^^3.5^ CFU/g, 10^^7^ CFU/g, and 10^^10^ CFU/g for the low, medium, and high groups, respectively, then multiplied by the number of grams of food in each group and summed, and later re-classified into low, medium, and high groups by <10^^8^ CFU, 10^^8^-10^^11^ CFU, and >10^^11^ CFU.

**Supplementary Table S4. Sensitivity analysis on the relationship between dietary intake of live microbes and Accidents Injuries mortality.**

|  | Crude model | |  | Adjusted Model | |
| --- | --- | --- | --- | --- | --- |
|  | HR (95%CI) | P value |  | 95%CI | P value |
| Low | ref |  |  | ref |  |
| Medium | 0.94(0.61,1.47) | 0.80 |  | 1.03(0.61,1.74) | 0.91 |
| High | 1.05(0.64,1.75) | 0.84 |  | 1.28(0.68,2.39) | 0.45 |
| P for trend | | 0.89 |  |  | 0.49 |
|  |  |  |  |  |  |
| Log-DILM | 1.01(0.91,1.13) | 0.84 |  | 1.07(0.93,1.23) | 0.36 |

Log-DILM: Log-(dietary intake of live microbes).

Data are presented as HR (95%CI).

Crude model was an unadjusted model.

Adjusted Model adjusted for age, sex, race, education, marital status, poverty-to-income ratio, Healthy Eating Index, total energy intake, smoking status, alcohol status, physical activity, BMI, hypertension, diabetes, CVD, cancer, and eGFR.

Dietary microbial counts were divided into three groups: low, medium, and high, with values of <10^7 CFU, 10^7-10^10 CFU, and >10^10 CFU respectively.
